# A Pilot Study for Inducing Chronic Heart Failure in Calves by Means of Oral Monensin

**Published:** 2010-03

**Authors:** Roula Zahr, Diyar Saeed, Hideyuki Fumoto, Tetsuya Horai, Shanaz Shalli, Tomohiro Anzai, Yoko Arakawa, Raymond Dessoffy, Jacquelyn Catanese, Alex Massiello, Kenneth N. Litwak, Kiyotaka Fukamachi

**Affiliations:** 1 *Department of Biomedical Engineering, Lerner Research Institute, Cleveland Clinic, Cleveland, Ohio, USA;*; 2 *Biological Resources Unit, Cleveland Clinic, Cleveland, Ohio, USA*

**Keywords:** animal models, bovine, heart failure, ionophores, hemodynamics

## Abstract

**Introduction:**

Heart failure remains a major cause of mortality in the United States, despite advancing technologies, newer methods of treatment, and novel devices. To evaluate such novel devices, a large-animal model of chronic heart failure is critical in carrying out preclinical animal studies.

**Methods:**

We evaluated the efficacy of oral monensin in inducing stable heart failure in five Jersey calves. Various doses of monensin were administered. Hemodynamics, pressure–volume loops, echocardiographic measurements, extent of tissue perfusion, and histopathologic data were recorded before and after induction of heart failure.

**Results:**

Responses were variable in the animals. One experiment showed a significant decrease in cardiac output within one week, associated with simultaneous increases in left atrial pressure, central venous pressure, and mean pulmonary artery pressure. Left ventricular pressure-volume loops showed that the slope of the end-systolic pressure-volume relation decreased markedly between the baseline and terminal study, suggesting a decrease in contractility. Echocardiographic studies indicated a decrease in ejection fraction. Histopathologic analysis in cardiac tissue showed extensive fibrosis and necrosis.

**Conclusion:**

We demonstrated the feasibility of inducing and maintaining severe yet stable heart failure for up to 3 weeks in a calf model by administration of oral monensin.

## INTRODUCTION

Despite optimal medical therapy, nearly 300,000 patients die from heart failure each year in the United States (1). About 10,000 qualify as transplant candidates, yet only about 2,200 heart transplants are performed every year (2). As a consequence, innovative surgical procedures, ventricular assist devices, and left ventricular remodeling techniques have emerged as the preferred technologies for treatment of end-stage heart disease (3, 4).

It is important to evaluate the efficacy of these new technologies in preclinical animal studies. Large-animal models, such as calves, are frequently used because they bear some similarities to human cardiac anatomy and physiology; such models are useful in overcoming device fitting problems, as well. A healthy calf model is appropriate for demonstrating device biocompatibility; however, stable large-animal models of chronic stable heart failure are needed to evaluate the efficacy of innovative therapeutic strategies against heart failure. One study evaluating monensin toxicosis in calves suggested that this species may provide a valuable model of inducible chronic heart failure as this model is effective without using invasive procedures (5). However, further characterization of this model is still necessary. In this pilot study, we evaluated the induction of chronic stable heart failure by means of multiple oral monensin dosages in calves.

## MATERIALS AND METHODS

This study was approved by the Cleveland Clinic’s Institutional Animal Care and Use Committee. All animals received humane care in compliance with the “Guide for the Care and Use of Laboratory Animals” prepared by the Institute of Laboratory Animal Resources, National Research Council, and published by the National Academy Press (revised 1996).

### Monensin Sodium

Monensin is a polyether ionophore antibiotic agent produced by fermentation of *Streptomyces cinnamonensis*. It is approved by the US Food and Drug Administration to treat ruminant coccidiosis and to improve milk production efficiency in dairy cows. However, in high doses, monensin causes cardiac toxicity, documented in the literature by accidental or intentional administration of higher than recommended doses of monensin to many species, including sheep (6), horses (7), buffaloes (8), swine (9), and calves (10–12). We used a “pure” form, Monensin, Sodium Salt 97% (Acros Organics N.V., Fair Lawn, New Jersey, USA) in our pilot study.

### Animal Preparation and Surgical Procedures

Five male Jersey calves (body weight 76.4 ± 8.5 kg) were used in this pilot study. Each animal was fasted for 12 h before surgery. Anesthesia was induced with an intramuscular injection of ketamine, with isoflurane given via mask inhalation. After endotracheal intubation, anesthesia was maintained with isoflurane (0.5–2.5%). A fluid-filled pressure monitoring line was inserted into the jugular vein to monitor central venous pressure (CVP). A median sternotomy approach was used for the first three experiments for the purpose of performing pressure-volume loop study, while a left thoracotomy was used for the next two experiments. A fluid-filled line was inserted into the pulmonary artery to monitor pulmonary artery pressure (PAP). A 28-mm Transonic flow probe (Transonic Systems, Inc, Ithaca, New York, USA) was placed around the ascending aorta to monitor cardiac output (CO). A fluid-filled line was placed within the left atrium for left atrial pressure (LAP) monitoring. An arterial fluid-filled line was connected to a side port of a carotid sheath to monitor arterial pressure (AoP). A conductance catheter with two Millar pressure sensors (SPC 562, Millar Instruments, Houston, Texas, USA) was inserted through the carotid sheath into the left ventricle (LV) to monitor LV pressure-volume (PV) loops in the first three experiments. Umbilical tapes were placed around the superior vena cava and inferior vena cava. A two-dimensional epicardial echocardiogram (2D EE) was performed to confirm the position of the conductance catheter, for calibration of the conductance catheter measurements, and to assess left and right ventricular functions at baseline. The superior and inferior vena cavae were transiently occluded using the umbilical tapes to obtain the LV PV loops under various preloads.

### Postoperative Management and Induction of Heart Failure

Postoperatively, the animals were allowed to recover in the chronic care unit (CCU), and standard postoperative care was given. Butorphanol (10–20 mg intravenously (I.V.) × every 12 h for 5 d, then PRN [“as needed”]) and flunixamine (100 mg I.V. × every 8 h for 5 d, then PRN) were administered for breakthrough pain. Gentamicin (160 mg I.V. × every 8 h) and cefazolin (2 g I.V. × every 6 h) were given postoperatively for 7 d.

After a 7-d recovery period, each calf was sedated with intravenous xylazine (6–8 mg). An oral-gastric tube was inserted, followed by a Corpak feeding tube, which was inserted through the gastric tube. A single dose of monensin (Acros Organics N.V., Fair Lawn, New Jersey, USA) suspended in 60 mL of normal saline solution was injected down the Corpak tube using a 60-mL syringe. After the initial dose, the syringe was flushed multiple times with normal saline.

Monensin blood plasma concentrations were measured 1 d after each dose was given. Levels of plasma renin activity, and the cardiac enzymes troponin T and creatine kinase-myocardial band (creatine kinase-MB) were measured before and then one week after each monensin administration. Successful induction of heart failure was defined as either a 20%–30% increase of left ventricular end-diastolic volume (EDV) above baseline, a 10–20 mm Hg decrease in mean systemic blood pressure below baseline, a 10 mm Hg increase in LAP and/or CVP above baseline, or a 20% decrease in CO below baseline.

After 5 wk of heart failure induction, a second surgical procedure was performed in the surviving calves to obtain terminal hemodynamic data. Epicardial echocardiogram and LV PV loops studies were performed and compared with baseline measurements.

### Hemodynamics and LV PV Loop Studies

Hemodynamic parameters were recorded hourly after the initial surgery by the animal monitor in the CCU and weekly via a data acquisition system (PowerLab, ADInstruments Inc., Mountain View, CA, USA). Data were stored on a hard disk for subsequent analysis by a laboratory computer. The following parameters were recorded: PAP (systolic, mean, and diastolic), AoP (systolic, mean, and diastolic), CVP and LAP through the fluid-filled catheters, heart rate via electrocardiogram, and CO by means of flow probe readings.

As we previously reported (13), conductance volumes were corrected using a two-point calibration based on echocardiographic measurements matching EDV and end-systolic volume (ESV) under steady-state conditions (14). LV performance was assessed by measuring the contractility demonstrated by the end-systolic pressure-volume relationship (ESPVR) (15, 16).

### Two-Dimensional Echocardiography

A Vivid 7 echocardiography machine (GE Medical, Milwaukee, Wisconsin, USA) coupled to a 4S transducer (multifrequency transducer) was used in this study. For both initial and terminal studies, 2D EEs were performed during the surgical procedures. Two-dimensional transthoracic echocardiograms were performed at a single time point before drug administration, and weekly thereafter. The 2D EEs were used to calculate LV volumes and ejection fraction (EF) using Simpson’s biplane method.

### Regional Blood Flow and Tissue Perfusion

Regional blood flow was measured by using labeled microspheres (BioPal™, BioPhysics Assay Laboratory, Inc., Worcester, MA, USA). BioPAL microsphere injection was performed once in the CCU before monensin administration to get a baseline value, then weekly after monensin administration. Microspheres were injected into the left atrium through the LAP monitoring line, while a reference blood sample was withdrawn through a syringe pump from the carotid artery. Tissue samples harvested at autopsy and blood samples of interest were sent to the BioPAL laboratory for processing and evaluation of the regional blood flow.

### Autopsy

At the end of each study, the animal was killed by rapid intravenous injection of pentobarbital (50 mg/kg) and potassium chloride (240 mEq). Following sacrifice, a thorough autopsy was performed, and histological specimens were harvested from the heart for routine hematoxylin-and-eosin staining study under light microscopy (magnification of 100× with a 10× ocular lens). Moreover, samples from the LV free wall, septum, RV free wall, and kidney were obtained for the analysis of regional blood flow with microspheres.

### Study Protocol

Table 1 summarizes the protocol followed for each of the five experiments we conducted. The experiment numbers refer to the chronological order in which they were performed. In experiment 1, the animal survived four doses of monensin; 25, 20, 40, and 60 mg/kg were administered, but no heart failure parameters were detected, and the animal was electively sacrificed 65 d after receiving the first dose. In experiment 3, the animal received two doses, a first dose of 60 mg/kg, and a second dose of 80 mg/kg given 12 days after administering the first one; signs and symptoms of heart failure were recorded, and the terminal study was conducted 3 weeks after the documented induction of chronic stable heart failure. In experiments 2, 4 and 5, each calf received only one dose of monensin (80, 60, and 40 mg/kg, respectively), and each experiment was terminated because of the unexpected death of the animal.

**Table 1:** Study protocol

Animal	Study parameters	Pre-Monensin	Dose number				Study duration	Cause of study termination
			1	2	3	4		

1	Dose (mg/kg)	-	25	20	40	60	-	Elective sacrifice
	PMD (days)	-	0	13	24	35	65	
	Monensin [ng/mL]	-	-	-	-	-	-	
	Plasma renin activity [μg/l/hr]	0.9	1.3	0.4	0.6	0.5	-	
	CK-MB [ng/ml]	-	-	-	-	0.6	-	
	Troponin T [ng/ml]	<0.01	<0.01	<0.01	<0.01	<0.01	-	
2	Dose (mg/kg)	-	80	-	-	-	-	Sudden death from cardiac arrhythmia
	PMD (days)	-	0	-	-	-	6	
	Monensin [ng/mL]	-	7.21	-	-	-	-	
3	Dose (mg/kg)	-	60	80	-	-	-	Elective sacrifice
	PMD (days)	-	0	12	-	-	34	
	Monensin [ng/mL]	-	N/A	5.05	-	-	-	
	Plasma renin activity [μg/l/hr]	0.7	0.7	1.0	-	-	-	
	CK-MB [ng/ml]	1.3	1.1	1.0	-	-	-	
	Troponin T [ng/ml]	<0.01	<0.01	<0.01	-	-	-	
4	Dose (mg/kg)	-	60	-	-	-	-	Severe refractory heart failure
	PMD (days)	-	0	-	-	-	4	
	Monensin [ng/mL]	-	6.75	-	-	-	-	
5	Dose (mg/kg)	-	40	-	-	-	-	Severe refractory heart failure
	PMD (days)	-	0	-	-	-	5	
	Monensin [ng/mL]	-	23.0	-	-	-	-	

Monensin [ng/mL], blood concentration 1 d after each dose; PMD, post first-monensin day; CK-MB, creatine kinase-myocardial band. Plasma renin activity, CK-MB, and troponin T levels were measured pre-monensin and 7 days after each dose administration.

## RESULTS

**Figure 1:**
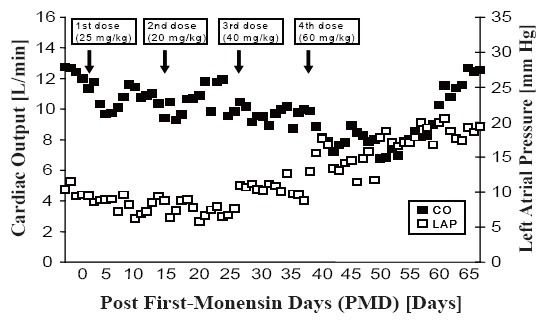
Changes in cardiac output (CO) and left atrial pressure (LAP) after four doses of oral monensin administration in animal 1.

Experiment 1: The initial monensin dose given was 25 mg/kg, based on results from a study at the University of Louisville (5). CO decreased from 12.0 ± 1.2 L/min to 9.7 ± 0.8 L/min over 4 d; however, it returned to baseline levels by the sixth day after the first dose of monensin had been given (the “post first dose of monensin day” [PMD]), as shown in Figure 1. In addition, there were no changes in LAP. Therefore, a second dose (20 mg/kg) was given on PMD 13, which resulted in a similar moderate drop in CO, followed by recovery within 1 week. A third dose (40 mg/kg) given on PMD 24 did not significantly affect hemodynamics. Therefore, a fourth dose of 60 mg/kg was given on PMD 35. Although CO decreased to 6.8 ± 0.8 L/min and LAP increased to 18 ± 3 mm Hg over 15 d, CO returned to baseline levels within 30 d of administration of the fourth dose (60 mg/kg). Changes in echocardiographic data on day 17 after administration of the fourth dose compared with measurements prior to administration of the same dose showed mild changes: in the right ventricle, EDV increased from 52 mL to 67 mL and ESV from 18 mL to 27 mL, and there was a minimal decrease in RV EF from 65% to 60%; in the left ventricle, there was no change in EDV, an increase in ESV from 24 mL to 35 mL, and a decrease in LV EF from 71% to 69%. The recorded decrease in CO was also demonstrated by a consistent decrease in tissue perfusion as measured by the microspheres method. Histopathologic study of the cardiac tissue showed hyperplastic changes in cell structure and cellular degeneration with resultant fibrosis.

**Figure 2:**
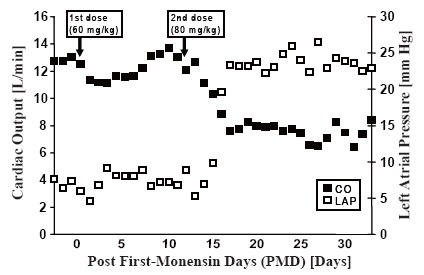
Changes in cardiac output (CO) and left atrial pressure (LAP) after two doses of oral monensin administration in animal 3.

**Figure 3:**
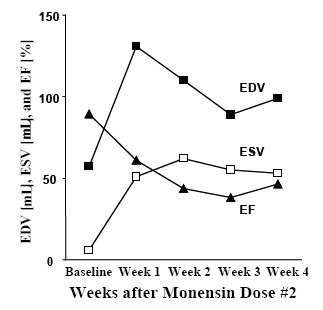
Changes in LV volumes and ejection fraction (EF) from echocardiography in animal 3.

**Figure 4:**
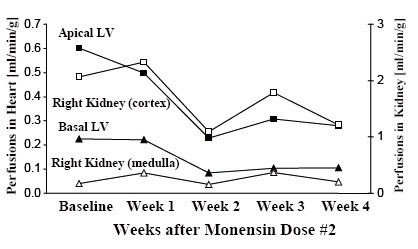
Tissue perfusions in the heart and kidney determined by BioPal™ microspheres in animal 3.

Experiment 3: The animal received an initial monensin dose of 60 mg/kg, which did not significantly affect hemodynamics (Figure 2). After a second dose of 80 mg/kg on PMD 12 resulting in serum levels as seen in Table 1, there was a dramatic decrease in CO from 12.0 ± 1.2 L/min to 7.9 ± 0.6 L/min, associated with simultaneous increases in LAP from 7 ± 3 mm Hg to 23 ± 4 mm Hg, CVP from 5 ± 2 mm Hg to 12 ± 3 mm Hg, and mean PAP from 22 ± 3 mm Hg to 39 ± 4 mm Hg; a decrease in mean AoP from 92 ± 17 mm Hg to 85 ± 8 mm Hg also was evident. The LV PV loops in this animal showed that the slope of the ESPVR decreased remarkably from 1.1 mm Hg/mL at baseline to 0.5 mm Hg/mL at the terminal study. Changes in echocardiographic data are shown in Figure 3. Results of a tissue blood perfusion study demonstrated a decrease in tissue perfusion in the heart and renal cortex that was consistent with the decrease in CO, as shown in Figure 4. Histopathologic changes in cardiac tissue were associated with extensive fibrosis, necrosis, and inflammation.

Experiments 2, 4, and 5: The animals died unexpectedly from various dosages of monensin. Higher concentrations of monensin were detected in the blood plasma in these calves (Table 1). The histopathologic examinations of the cardiac tissues from these animals showed multiple areas of muscular degeneration and necrosis.

The levels of plasma renin activity, troponin T, and creatine kinase-MB were only measured in the surviving animals (1 and 3) one week after each dose administration. As shown in Table 1, there were no changes in the levels of plasma renin activity, troponin T, or creatine kinase-MB.

## DISCUSSION

In a previous study, elevation in cardiac troponin I was reported after administration of sodium monensin in horses (17). In our study, there were no remarkable changes in the levels of plasma renin activity, cardiac troponin T, or creatine kinase-MB (Table 1). A previous study on alterations in myocardial cells on the ultrastructural level after monensin administration showed sarcoplasmic vacuolation from mitochondrial swelling and lipid accumulation or myocardial necrosis (11).

In this study, we demonstrated the feasibility of inducing and maintaining stable yet severe heart failure for up to 3 weeks in a calf by oral administration of monensin. The recorded changes in hemodynamic parameters in experiment 3 after dose 2, demonstrated by a marked decrease in CO and mean AoP accompanied by a similar increase in PAP, LAP and CVP, are consistent with heart failure and were stable for 3 weeks until the animal was electively sacrificed. The decrease in the slope of the ESPVR suggested a marked decrease in LV contractility. Measurements from experiment 3’s LV PV loops, hemodynamics, echocardiography, colored microspheres tissue perfusion study, and pathologic examination all confirmed severe, persistent cardiomyopathy. The model was associated with structural changes consistent with LV remodeling (18), reduction in LV performance measured by means of load-independent methods, impaired hemodynamics and diminished tissue perfusion.

Our results demonstrated large variability in response to oral monensin among the five calves, ranging from the absence of heart failure with 60 mg/kg monensin in the first experiment to severe heart failure with 40 mg/kg monensin in experiment 5. As shown in Table 1, the blood concentration of monensin after 1 d of administration was higher in the animals that died prematurely (in experiments 2, 4, and 5) than in experiment 3, which demonstrated stable, chronic heart failure. Monensin blood concentration, however, showed no correlation with the amount of monensin administered. This variability in blood concentration is thought to be due to variability in drug absorption rate from the alimentary tract in ruminants when monensin is given orally. Further clarification of monensin’s pharmacological mechanisms in cattle is important to control serum levels of monensin after oral administration in these animals.

There are several available large-animal models of heart failure, including models of tachycardia-induced heart failure (i.e., rapid ventricular pacing (19)), myocardial damage (i.e., coronary artery ligation (20), microembolism (21), and toxic cardiomyopathy (22)), pressure overload (i.e., aortic banding (23)), and volume overload (i.e., arteriovenous anastomosis (24), and mitral valve regurgitation (25)). There are also drug-induced models using drugs with negative inotropic effects (26). Each model has its respective shortcomings, such as reversibility, toxicity to the rest of the body, applicability only in acute studies, and the need for surgical intervention and/or significant technical skills. Some of these models reproduce the neurohormonal changes of naturally occurring congestive heart failure, whereas others better reproduce the remodeling that occurs during chronic heart failure.

The model of chronic heart failure induced by monensin is more reliable than other models of drug-induced heart failure, since it overcomes the disadvantages of reversibility and the need for continuous drug infusion that characterizes most of these models, as in using drugs with negative inotropic effects (26). On the other hand, monensin administration precludes the severe systemic toxic effects of doxorubicin and adriamycin, which has compelled researchers to directly apply these drugs through an angiocatheter to the coronary artery (27, 28) - a technique that is difficult to perform in calves, primarily because of the anatomy and the body volume of these animals, not to mention the invasiveness and anaesthesia requirements involved in such procedure, compared to simple administration of oral monensin in our pilot study.

The ideal animal model of congestive heart failure should be able to mimic the progression of naturally occurring human congestive heart failure syndrome, characterized by a complex alteration in hemodynamics and geometry of the heart, as well as reflexes in the sympathetic nervous system, the cardiac endocrine system, and the renin-angiotensin system.

### Study Limitations

The main limitation in this study was the small number of animals used. Although oral monensin was successful in inducing chronic stable heart failure in one animal, there was no uniform protocol to induce a reliable reproducible model among all the animals in this study.

## CONCLUSION

Although no conclusion can be presented, we have successfully administered monensin orally in our study to induce severe chronic stable heart failure; the main problem was to control the monensin blood concentration administering monensin this way probably due to a large variability of monensin absorption from the alimentary tract in cattle. Future investigations are required to further characterize the heart failure induced by monensin and the resulting impact on cardiac tissue and cardiac enzymes.
